# A case report of vaccine-induced immune thrombotic thrombocytopenia (VITT) with genetic analysis

**DOI:** 10.3389/fcvm.2023.1189320

**Published:** 2023-05-19

**Authors:** Daniela P. Mendes-de-Almeida, Fernanda S. G. Kehdy, Remy Martins-Gonçalves, Joanna Bokel, Eduarda Grinsztejn, Patrícia Mouta Nunes de Oliveira, Maria de Lourdes de Sousa Maia, Brenda Hoagland, Sandra Wagner Cardoso, Beatriz Grinsztejn, Marilda M. Siqueira, Pedro Kurtz, Patricia T. Bozza, Cristiana C. Garcia

**Affiliations:** ^1^ Department of Hematology, Evandro Chagas National Institute of Infectious Diseases, Fundação Oswaldo Cruz (FIOCRUZ), Rio de Janeiro, Brazil; ^2^ Department of Medical Affairs, Clinical Studies, and Post-Registration Surveillance (DEAME), Institute of Technology in Immunobiologicals/Bio-Manguinhos, Fundação Oswaldo Cruz (FIOCRUZ), Rio de Janeiro, Brazil; ^3^ Research Center, Instituto Nacional de Câncer (INCA), Rio de Janeiro, Brazil; ^4^ Laboratório de Hanseníase, Instituto Oswaldo Cruz, Fundação Oswaldo Cruz (FIOCRUZ), Rio de Janeiro, Brazil; ^5^ Laboratory of Immunopharmacology, Instituto Oswaldo Cruz, Fundação Oswaldo Cruz (FIOCRUZ), Rio de Janeiro, Brazil; ^6^ Onco-Hematology Unit, Clínica São Vicente, Rio de Janeiro, Brazil; ^7^ Department of Medicine, Hematology and Oncology Division, University Hospitals, Case Western University, Cleveland, OH, United States; ^8^ Laboratory of Clinical Research on STD/AIDS, Evandro Chagas National Institute of Infectious Diseases Oswaldo Cruz Foundation (FIOCRUZ), Rio de Janeiro, Brazil; ^9^ Laboratório de Vírus Respiratórios, Exantemáticos, Enterovírus e Emergências Virais, Instituto Oswaldo Cruz, Fundação Oswaldo Cruz (FIOCRUZ), Rio de Janeiro, Brazil; ^10^ Intensive Care Department, Instituto D’Or de Pesquisa e Ensino, Rio de Janeiro, Brazil; ^11^ Grupo Integrado de Pesquisas em Biomarcadores, Instituto René Rachou, Fundação Oswaldo Cruz (FIOCRUZ-Minas), Belo Horizonte, Brazil

**Keywords:** vaccine-induced thrombotic thrombocytopenia, ChAdOx1 nCoV-19 vaccine, genetic predisposition, polymorphisms, anti-PF4 antibodies, VITT

## Abstract

The emergence of the rare syndrome called vaccine-induced immune thrombocytopenia and thrombosis (VITT) after adenoviral vector vaccines, including ChAdOx1 nCov-19, raises concern about one's predisposing risk factors. Here we report the case of a 56-year-old white man who developed VITT leading to death within 9 days of symptom onset. He presented with superior sagittal sinus thrombosis, right frontal intraparenchymal hematoma, frontoparietal subarachnoid and massive ventricular hemorrhage, and right lower extremity arterial and venous thrombosis. His laboratory results showed elevated D-dimer, C-reactive protein, tissue factor, P-selectin (CD62p), and positive anti-platelet factor 4. The patient's plasma promoted higher CD62p expression in healthy donors' platelets than the controls. Genetic investigation on coagulation, thrombophilia, inflammation, and type I interferon-related genes was performed. From rare variants in European or African genomic databases, 68 single-nucleotide polymorphisms (SNPs) in one allele and 11 in two alleles from common SNPs were found in the patient genome. This report highlights the possible relationship between VITT and genetic variants. Additional investigations regarding the genetic predisposition of VITT are needed.

## Introduction

Mass vaccination against SARS-CoV-2 was the main measure to mitigate hospitalizations, long-term health outcomes, and death due to the COVID-19 pandemic. However, after millions of doses were administered, reports of a very rare syndrome called vaccine-induced immune thrombocytopenia and thrombosis (VITT) began to rise (1). The hallmark features are thrombocytopenia, thrombosis within 5–30 days of adenoviral SARS-CoV-2 vaccination, with strikingly elevated levels of D-dimer, hypofibrinogenemia, and positive antibodies against platelet factor 4 (PF4) (2). The clinical presentation depends on the thrombosis site. The typical targets are the cerebral venous sinus or splanchnic vein, arterial, or multiple beds (1–3).

The underlying mechanism of the syndrome is similar to heparin-induced thrombocytopenia (HIT), with the formation of aggregates of PF4 and the ChAdOx1 adenovector in an inflammatory environment induced by vaccination, with subsequent generation of high-avidity anti-PF4 IgG, which triggers platelet activation, prothrombotic cascade, and release of neutrophil extracellular traps (NETs) (4). However, the interplay between anti-PF4 antibodies and platelet activation is complex. A longitudinal study showed a slight but transient thrombin generation after ChadOx1 nCov-19. In addition, 19.6% (12/61) samples were positive for anti-PF4 before vaccination and 3.2% (2/61) were considered strong. Low titers of anti-PF4 remained unchanged after vaccination, and no seroconversion was detected. No thrombotic events occurred in this study (5).

Differences in incidence among countries and detection of oligoclonal anti-PF4 antibodies raised the suspicion of a genetic predisposition in VITT (6, 7). Individual factors like genetic ancestry might play a critical role in disease pathogenesis. Here, we report a fatal case of a Brazilian male who developed VITT 4 days after vaccination with ChAdOx1 nCov-19, its platelet activation profile, and genetic analysis.

## Case description

A 56-year-old white man with a history of essential hypertension controlled with atenolol received the first ChAdOx1 nCov-19 vaccine in early May 2021. Four days after vaccination, he developed fever, malaise, and persistent headache. On the fifth day following vaccination, he presented with nausea, vomiting, fall from his height, generalized skin rash on the lower limbs, and ecchymosis. He was promptly admitted. His platelet count was 17,000/mm^3^ (150,000–450,000/mm^3^), D-dimer 41,000 ng/ml (<500 ng/ml), and fibrinogen 121 mg/dl (200–400 mg/dl). Peripheral smear showed no platelet clumps or schistocytes. Brain computed tomography (CT) examination identified right frontal heterogeneous intraparenchymal hematoma, measuring approximately 6.4 cm × 5.2 cm × 4.8 cm with a thin hypodense halo, causing mass effect with a local reduction in the amplitude of the sulci, compression over the right lateral ventricle resulting in contralateral deviation of midline structures by 0.7 cm, in addition to areas of bilateral frontoparietal subarachnoid hemorrhage. He also presented with a massive ventricular hemorrhage filling in the right lateral ventricle, the posterior horn of the left lateral ventricle, and the fourth ventricle, and bleeding in the right Sylvian cistern and perimesencephalic cistern. There was no evidence of aneurysmal dilatation. The CT angiogram the following day identified thrombosis in the superior sagittal sinus. Also, a lower extremities Doppler ultrasound showed right arterial and venous thrombosis. He underwent urgent neurosurgery for hematoma drainage and decompressive craniectomy. A few hours after the procedure, he developed new bleeding and bilateral cerebral edema and received plasma, cryoprecipitate, fibrinogen concentrate, platelet transfusions, and 70 g (1 g/kg) intravenous immunoglobulin. Despite all measures, the patient died with refractory intracranial hypertension on day 13 after vaccination.

## Laboratory and clinical investigation

Patient’s relatives and healthy unvaccinated controls provided written informed consent approved by the local Ethics Committee for clinical and laboratory investigations (CAAE #68118417.6.0000.5248 and #48532621.8.0000.5262, respectively). SARS-CoV-2 RT-PCR of the nasopharyngeal swab and serology to dengue, Chikungunya, Zika, HIV, hepatitis B and C, Cytomegalovirus (CMV), Epstein-Barr virus (EBV), toxoplasmosis, and rubella were negative. Relatives denied any past COVID-19 infection or heparin exposure. There was no personal or family history of thrombosis or miscarriages.

**Figure 1:**
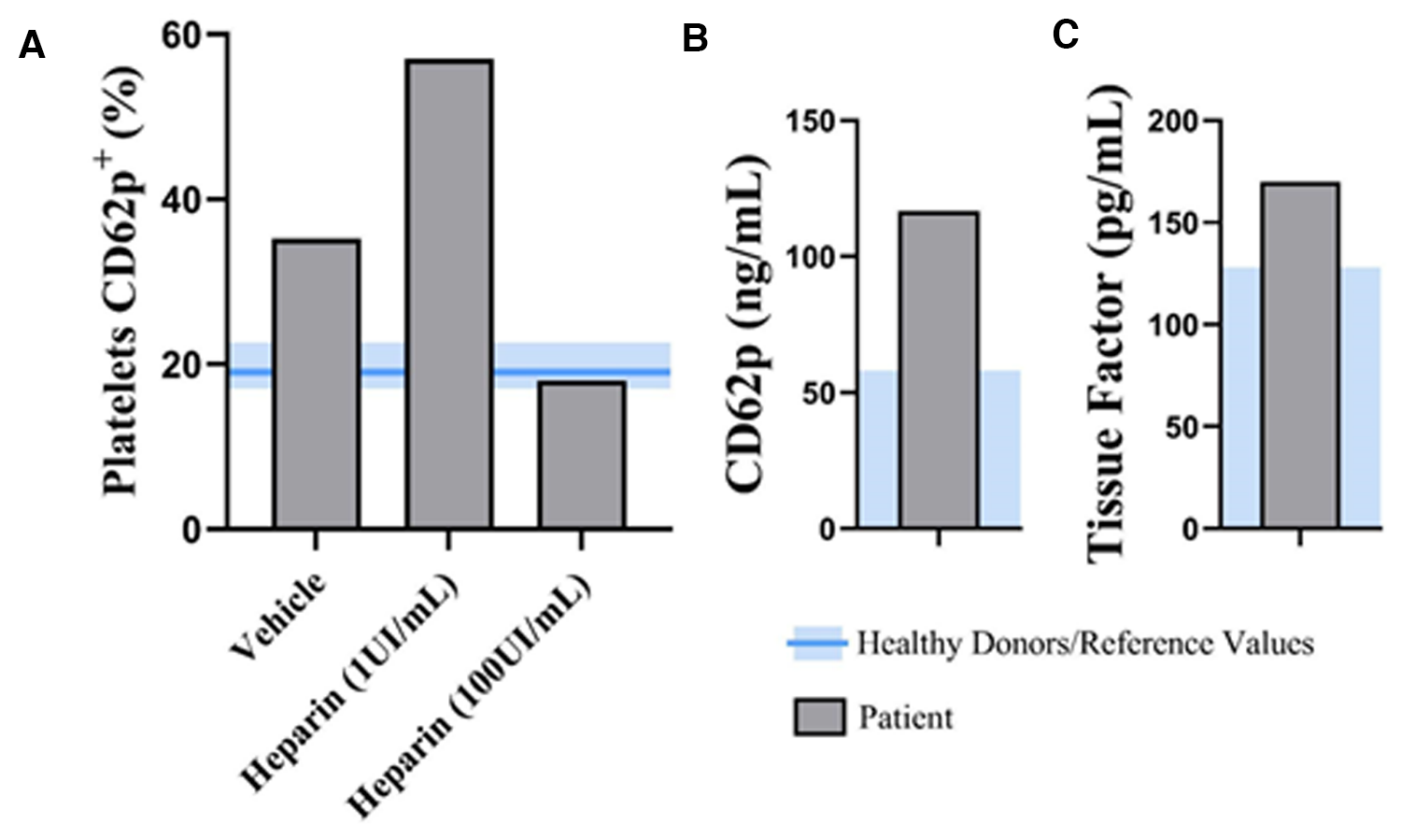
Functional VITT test, markers of platelet activation and coagulation. (**A**) A flow cytometry-based assay to detect platelet-activating antibodies was performed according to Handtke et al. Levels of CD62p surface expression in healthy donor's platelets stimulated with plasma from the patient in the presence of low (1 UI/ml) and high (100 UI/ml) concentrations of heparin. Plasmatic levels of CD62p (**B**) and tissue factor (**C**). The blue area represents the reference range of healthy donors. VITT, vaccine-induced immune thrombocytopenia and thrombosis;

IgG anti-PF4 antibodies were detected with a 3.33 optical density (reference ≤ 0.4). A flow cytometry-based assay to detect platelet-activating antibodies was performed according to Handtke et al. (8) (Figure 1A). When added to healthy donor platelets, patient plasma elicited increased expression of CD62p to a greater extent than plasma from healthy heterologous donors. However, in the presence of high concentrations of heparin, which can destabilize PF4/adenovector aggregates due to its higher affinity to PF4, platelet activation levels were reduced to control levels, confirming the presence of platelet-activating immunocomplexes in the patient's plasma. Elevated plasmatic levels of CD62p, released by activated platelets and endothelial cells, and of tissue factor (TF, coagulation factor III), the primary activator of the extrinsic pathway of the coagulation cascade, corroborate the extensive platelet activation and clot formation (Figures 1B,C). Additional laboratory results are characterized in Table 1. The results of other blood tests were unremarkable except for increased alanine aminotransferase, C-reactive protein, IL-1β, and caspase-1. Antinuclear antibodies, anti-cardiolipin IgG and IgM, lupus anticoagulant, and beta-2 glycoprotein 1 IgG were not detected.

**Table 1:** Patient’s laboratory data.

Laboratory test	Reference range	01/20/21	05/14/21	05/16/21^a^
Hemoglobin (g/dl)	12.0–16.0	16.1	10.9	6.1
Platelet count (per mm^3^)	150,000–450,000	207,000	17,000	17,000
Leucocytes (per mm^3^)	4,500–11,000	6,860	8,700	9,700
D-dimer (ng/ml)	<500		41,000	97,016
Fibrinogen (mg/dl)	200–400	264	121	
Activated partial thromboplastin time (rel)	<1.25		0.94	
International normalized ratio	0.8–1.2		1.31	
C-reactive protein (mg/dl)	<0.3			18.40
Aspartate aminotransferase (U/l)	15–37	21		176
Alanine aminotransferase (U/l)	6–45	48		79
PCR Sars-Cov-2				Not detected
Anti-heparin/PF4 ELISA (OD)	≤0.4		3.33	
Tissue factor (pg/ml)	90–150			170
P-selectin (pg/ml)	23–59			116.6
IL-18 (pg/ml)	≤650			493.6
IL-1β (pg/ml)	≤8			135.9
Caspase-1 (pg/ml)	≤148			226.6
PF4 (ng/ml)	50–155			63.2

OD, optical density.

^a^ Functional assay.

We performed genetic analysis using the Axiom™ Human Genotyping SARS-CoV-2 Research Array, which genotypes more than 870,000 single-nucleotide polymorphisms (SNPs) in the human genome. The first strategy consisted of screening mutations in 232 autosomal genes essential for thrombotic syndromes, to inflammatory disorders, and related to type I interferon (IFN) signaling (Table 2). Aiming to select rare variants, the minor allele frequency up to 0.01 in European or African populations was settled as a cutoff. Six thousand four hundred sixty-six related SNPs were present in the array, and 5,953 are described in the 1KGP database. From the selected SNPs, 689 and 845 rare putative variants were found in databases of African and European populations, respectively. Among them, 68 were found in heterozygosity in the patient. From these, seven SNPs have been studied in clinical conditions; four were considered benign; one likely benign; one, rs116667976, in the Factor XI gene (*F11*) with conflicting interpretations of pathogenicity (9); and the last one, rs2884737, in the *VKORC1* gene, associated with Warfarin drug response. A descriptive analysis of the SNPs is depicted in Table 3.

**Table 2:** List of autosomal genes and number of SNPs investigated in the case of VITT.

Gene	SNPs on HGSRA	SNPs in 1KGP	SNPs with MAF <0.01 in AFR	SNPs with MAF <0.01 in EUR	Case's SNPs in Heterozygosity with MAF < 0.01 in AFR or EUR
ABCC4	86	84	7	15	1
ABCG5	7	7	0	0	0
ABCG8	16	16	0	0	0
ABO	101	49	3	4	0
ACE	136	108	5	40	0
ACTB	23	23	4	0	0
ACTN1	66	66	6	2	0
ADA	15	14	1	0	1
ADAMTS13	10	10	4	0	0
AIM2	34	34	1	8	1
ANKRD26	9	9	1	0	0
ANO6	60	56	10	8	1
AP3B1	84	81	11	6	2
AP3D1	31	31	3	4	0
APOA5	5	5	1	0	0
ARPC1B	1	1	0	0	0
BAZ1B	23	23	1	1	0
BLOC1S3	1	1	0	0	0
BLOC1S6	7	7	2	1	1
C1S	9	0	0	0	0
C2	54	43	7	6	0
C3	60	60	3	6	0
C3AR1	8	0	0	0	0
C4BPA	22	22	2	2	2
C5	17	17	2	2	1
C5AR1	17	17	4	3	0
C9	22	22	3	3	0
CADM1	57	57	5	9	0
CALR	6	4	1	2	0
CAMP	7	7	1	4	0
CARD8	31	31	4	6	0
CASP1	4	4	0	0	0
CASP4	5	5	1	0	0
CASP5	9	9	0	4	0
CD14	6	5	1	0	0
CD163	10	1	0	0	0
CD27	1	1	0	0	0
CD46	7	7	1	0	0
CD70	19	19	2	2	1
CDC42	15	15	1	0	0
CETP	41	41	2	1	0
CFB	26	23	9	6	2
CFD	12	12	0	3	0
CFH	30	29	2	2	0
CFHR1	1	1	0	0	0
CFHR2	1	1	0	0	0
CFHR3	5	5	0	1	0
CFHR4	4	4	2	0	0
CFHR5	7	7	2	3	0
CFI	10	9	0	2	0
CHI3L1	22	22	2	0	0
CORO1A	2	2	0	1	0
CPB2	12	12	0	1	0
CRP	67	67	4	7	0
CST1	15	15	0	1	0
CST4	18	17	3	1	0
CTPS1	41	41	5	5	0
CXCL8	8	8	0	1	0
CYCS	37	37	4	4	1
CYP4V2	8	8	0	1	0
DGKE	10	10	2	0	0
DIAPH1	8	8	2	0	0
DTNBP1	80	79	9	13	0
EDEM2	6	6	0	1	0
ETV6	195	193	17	32	4
FADD	1	1	0	0	0
FAS	12	12	1	0	1
FASLG	38	38	2	3	0
FCGR2A	18	18	4	2	0
FERMT3	3	3	1	2	0
FGA	15	10	1	1	0
FGB	14	11	0	1	0
FGG	11	9	0	2	0
FII	8	5	1	2	1
FLI1	56	56	2	4	0
FV	61	47	6	8	1
FVII	27	14	4	3	0
FX	26	12	2	2	0
FXI	31	10	0	1	1
FXII	11	5	1	1	0
GATA2	6	6	1	0	0
GCKR	14	14	0	1	0
GDF15	14	14	2	0	0
GFI1B	56	56	3	7	0
GGCX	21	10	1	1	0
GNE	9	9	0	2	0
GP1BA	11	5	1	2	0
GP6	28	26	2	2	0
GP9	5	5	1	2	0
GRN	35	14	1	4	1
HABP2	22	22	0	3	0
HAVCR2	8	8	1	0	0
HIVEP1	24	23	2	2	0
HOXA11	1	1	0	0	0
HPS1	10	10	1	1	1
HPS3	14	14	2	2	0
HPS4	22	22	4	1	1
HPS5	7	7	1	0	0
HPS6	2	2	0	0	0
HRG	9	9	1	3	0
IFI16	23	22	3	3	0
IFIH1	29	21	11	10	1
IFNA1	9	7	1	4	0
IFNA10	1	0	0	0	0
IFNA13	2	1	0	1	0
IFNA14	6	6	6	3	1
IFNA16	6	6	2	2	0
IFNA17	3	0	0	0	0
IFNA2	5	5	2	3	0
IFNA21	6	6	2	1	0
IFNA4	4	3	3	2	0
IFNA5	14	13	5	9	0
IFNA6	6	6	2	4	0
IFNA7	4	4	3	1	1
IFNA8	4	3	0	1	0
IFNAR1	17	14	6	8	1
IFNAR2	44	33	11	9	2
IFNB1	11	8	4	4	0
IFNE	2	2	0	1	0
IFNK	10	8	5	3	0
IFNW1	18	13	4	4	0
IKZF5	3	3	0	0	0
IL10	14	13	3	2	0
IL18	8	8	1	0	0
IL1B	9	9	0	0	0
IL2RA	42	42	9	7	2
IL6	22	21	3	0	1
IRAK4	6	6	2	1	0
IRF7	9	2	1	1	0
IRF9	3	3	0	1	0
ITGA2B	9	5	1	0	0
ITGB3	34	25	1	5	1
ITIH3	7	7	1	0	0
ITK	102	101	10	4	1
JAK1	48	43	28	9	3
JAK2	36	33	6	4	0
KDSR	8	8	0	1	0
KIF25	22	22	0	2	0
KLKB1	14	14	0	1	0
KNG1	24	15	1	0	0
LAMP1	15	13	1	5	0
LBP	24	23	1	3	0
LCN2	9	9	1	0	0
LIPA	67	65	10	15	3
LMAN1	13	10	4	1	0
LRG1	8	8	0	0	0
LYST	39	35	4	4	2
MASP2	7	7	0	0	0
MCFD2	7	7	0	1	0
MECOM	343	342	27	21	1
MEFV	19	18	5	4	0
MMACHC	1	1	0	0	0
MMP9	16	13	1	6	0
MPL	1	1	0	1	0
MPO	13	13	0	2	0
MTHFR	13	12	0	1	0
MVK	2	2	1	1	0
MYD88	1	1	0	0	0
MYH9	102	99	12	13	0
NAP1L4	25	24	4	2	0
NAT8B	2	2	1	0	0
NBEA	95	95	11	9	2
NBEAL2	4	4	1	0	0
NEK7	93	93	9	12	1
NLRC4	5	5	1	0	0
NLRP1	37	36	1	6	0
NLRP3	43	43	1	4	0
NLRP6	9	9	0	0	0
NLRP7	52	51	2	7	0
NR1I2	25	20	0	2	0
PDGFA	13	13	0	0	0
PDGFB	67	65	8	7	0
PF4	16	16	2	3	0
PLA2G4A	95	95	10	8	0
PLAT	21	18	3	4	0
PLAU	4	4	0	1	0
PLG	18	12	0	4	1
PNP	25	25	0	6	0
PRG4	5	5	1	2	0
PROC	26	8	0	1	0
PROCR	8	7	0	1	0
PROSI	19	15	2	6	0
PTGS1	24	21	4	4	0
PTX3	1	1	0	0	0
PYCARD	1	1	0	0	0
RAB27A	24	24	1	2	0
RASGRP2	8	5	1	2	0
RGS7	237	235	21	35	3
RUNX1	502	496	51	59	4
S100A8	6	6	2	1	0
S100A9	4	4	0	0	0
SAA1	9	9	0	0	0
SAA2	1	1	0	3	0
SELP	25	25	0	3	0
SERPINA 10	8	8	2	3	0
SERPINA1	37	35	3	4	0
SERPINA3	17	17	1	1	0
SERPINC1	11	8	1	4	0
SERPIND1	14	12	4	2	0
SERPINE1	23	21	4	1	0
SERPINF2	8	8	1	1	0
SLC44A2	30	29	4	2	1
SLC7A7	26	25	1	1	1
SLFN14	9	9	0	0	0
SRC	62	62	9	3	0
STAB2	78	78	12	3	2
STAT1	30	25	10	8	0
STIM1	35	35	3	5	0
STX11	49	49	2	13	0
STXBP2	19	19	3	6	0
STXBP5	20	19	3	3	0
TBK1	52	44	12	17	1
TBXA2R	10	9	1	1	0
TBXAS1	85	75	10	7	0
TC2N	18	18	0	0	0
THBD	18	18	1	4	0
THPO	7	7	0	0	0
TICAM1	23	22	6	7	0
TLR3	76	65	3	15	2
TNFRSF1A	8	7	0	1	0
TPM4	37	37	3	6	0
TRPM7	20	20	3	2	1
TSPAN15	23	23	4	4	1
TUBB1	16	10	2	3	0
TYK2	20	14	6	8	0
UNC13D	4	4	0	0	0
UNC93B1	29	27	3	11	0
VIPAS39	6	6	0	1	1
VKORC1	18	10	2	1	1
VPS33B	11	10	1	3	0
VWF	112	94	10	16	0
WT1	87	85	6	6	1
Total	6,466	5,953	689	845	68

VITT, vaccine-induced immune thrombocytopenia and thrombosis; SNPs, single-nucleotide polymorphism; HGSRA, human genotyping SARS-CoV-2 research array; 1KGP, 1000 genomes project; MAF, minor allele frequency; AFR, African population; EUR, European population.

**Table 3:** Annotation of rare SNPs found in heterozygosity and common SNPs in homozygosity (gray) in the patient.

Gene	Variant (VCF)	Chr	rsID	Genecode comprehensive category	Genecode comprehensive info	Clinical significance	Disease name	Review status	SIFTcat	PolyPhen cat	AFR 1KGP MAF	EUR 1KGP MAF	Patient genotype
AIM2	1-159092646-G-A	1	rs2518564	Intronic	AIM2	NA	NA	NA	NA	NA	0.010	0.808	GA
C4BPA	1-207103339-G-A	1	rs61815046	Upstream	C4BPA	NA	NA	NA	NA	NA	0.007	0.172	AG
C4BPA	1-207158980-G-A	1	rs76181153	Intergenic	C4BPA(dist = 14,008), AL445493.2(dist = 20,316)	NA	NA	NA	NA	NA	0.001	0.079	AG
F5	1-169522317-G-A	1	rs2420371	Intronic	F5	NA	NA	NA	NA	NA	0.001	0.064	GA
LYST	1-235853701-G-A	1	rs34341762	Intronic	LYST	NA	NA	NA	NA	NA	0.000	0.011	AG
LYST	1-235824437-A-G	1	rs35753830	Intronic	LYST	NA	NA	NA	NA	NA	0.010	0.223	AG
NEK7	1-198091159-C-T	1	rs72749413	Intergenic	LHX9(dist = 155681), NEK7(dist = 65,835)	NA	NA	NA	NA	NA	0.001	0.106	TC
RGS7	1-241059620-G-A	1	rs183029590	Intronic	RGS7	NA	NA	NA	NA	NA	0.004	0.061	AG
RGS7	1-241036695-T-C	1	rs538423	Intronic	RGS7	NA	NA	NA	NA	NA	0.005	0.406	CT
RGS7	1-241243124-C-A	1	rs72760521	Intronic	RGS7	NA	NA	NA	NA	NA	0.010	0.105	AC
JAK1	1-64963772-G-T	1	rs116528404	Intronic	JAK1	NA	NA	NA	NA	NA	0,002	0,016	TG
JAK1	1-64838867-T-C	1	rs310242	Intronic	JAK1	NA	NA	NA	NA	NA	0,000	0,134	TC
JAK1	1-64912220-T-C	1	rs72675483	Intronic	JAK1	NA	NA	NA	NA	NA	0,000	0,136	CT
IFIH1	2-162260675-G-T	2	rs17713557	Intergenic	FAP(dist = 17,219), IFIH1(dist = 6,399)	NA	NA	NA	NA	NA	0,003	0,047	TG
MECOM	3-169102384-A-G	3	rs79129760	Intronic	MECOM	NA	NA	NA	NA	NA	0.004	0.033	GA
F11	4-186284272-G-A	4	rs116667976	Intronic	F11	Conflicting interpretations of pathogenicity	Hereditary factor XI deficiency disease; not provided	Criteria provided, conflicting interpretations	NA	NA	0.000	0.003	AG
TLR3	4-186102925-C-T	4	rs62347994	Intergenic	TLR3(dist = 14,856), FAM149A(dist = 1,494)	NA	NA	NA	NA	NA	0,002	0,124	TC
TLR3	4-185984081-A-G	4	rs78642332	Intergenic	SORBS2(dist = 27,429),RNU4-64P(dist = 42,375)	NA	NA	NA	NA	NA	0,096	0,001	GA
AP3B1	5-78050187-C-T	5	rs252800	Intronic	AP3B1	NA	NA	NA	NA	NA	0.007	0.172	TC
AP3B1	5-78257979-C-A	5	rs10474531	Intronic	AP3B1	NA	NA	NA	NA	NA	0.004	0.336	AC
ITK	5-157202838-C-T	5	rs111782388	ncRNA intronic	AC010609.1	NA	NA	NA	NA	NA	0.002	0.100	
CFB	6-31947158-T-C	6	rs1048709	Exonic/synonymous SNV	AL645922.1,CFB	Benign	Macular degeneration; Complement component 2 deficiency; Atypical hemolytic-uremic syndrome 4; Complement factor B deficiency; not provided	Criteria provided, multiple submitters, no conflicts	NA	NA	0.009	0.152	TC
CFB	6-31946896-C-T	6	rs13194698	Intronic	AL645922.1,CFB	NA	NA	NA	NA	NA	0.002	0.009	TC
PLG	6-160722602-A-G	6	rs4252121	Intronic	PLG	Benign	Not provided	Criteria provided, single submitter	NA	NA	0.325	0.009	GA
CYCS	7-24992255-A-G	7	rs17232369	Intergenic	OSBPL3(dist = 10,621), CYCS(dist = 127,836)	NA	NA	NA	NA	NA	0.003	0.108	GA
IL6	7-22727814-A-G	7	rs2069832	Intronic	IL6	NA	NA	NA	NA	NA	0.001	0.411	AG
C5	9-121006922-C-T	9	rs17611	Exonic/nonsynonymous SNV	C5	Benign	Not specified; not provided	Criteria provided, multiple submitters, no conflicts	tolerated	benign	0.007	0.458	TC
IFNA7	9-21203292-A-G	9	rs117970353	Intergenic	IFNA7(dist = 1,087), IFNA10(dist = 2,889)	NA	NA	NA	NA	NA	0,000	0,014	GA
IFNA14	9-21254456-G-A	9	rs10115240	Intergenic	IFNA14(dist = 14,465), IFNA5(dist = 49,870)	NA	NA	NA	NA	NA	0,000	0,019	GA
FAS	10-88997863-A-G	10	rs9658706	Intronic	FAS	NA	NA	NA	NA	NA	0.001	0.097	GA
HPS1	10-98426622-A-C	10	rs12570988	Intronic	HPS1	NA	NA	NA	NA	NA	0.132	0.003	CA
IL2RA	10-6012120-T-C	10	rs41290329	UTR3	IL2RA(ENST00000379959.7:c.*752A > G, ENST00000379954.5:c.*752A > G)	Likely benign	Interleukin 2 receptor, alpha, deficiency of	Criteria provided, single submitter	NA	NA	0.001	0.010	TC
IL2RA	10-5990954-G-A	10	rs41294605	Intergenic	IL15RA(dist = 12,767), IL2RA(dist = 19,735)	NA	NA	NA	NA	NA	0.002	0.110	AG
LIPA	10-89244146-C-T	10	rs12240489	Intronic	LIPA	NA	NA	NA	NA	NA	0.011	0.089	TC
LIPA	10-89334037-G-A	10	rs59564102	Intronic	IFIT3, LIPA	NA	NA	NA	NA	NA	0.003	0.079	AG
LIPA	10-89218513-T-C	10	rs6586174	Intronic	LIPA	NA	NA	NA	NA	NA	0.007	0.214	TC
TSPAN15	10-69470965-C-T	10	rs78150807	Intronic	TSPAN15	NA	NA	NA	NA	NA	0.008	0.189	TC
F2	11-46739206-G-A	11	rs3136516	Intronic	F2	Benign	Thrombophilia due to thrombin defect; not provided	Criteria provided, single submitter	NA	NA	0.006	0.523	AG
WT1	11-32262490-T-C	11	rs11031673	Intergenic	AL078612.2(dist = 118,767), WT1(dist = 125,285)	NA	NA	NA	NA	NA	0.005	0.194	CT
ANO6	12-45400648-G-T	12	rs4768609	Intronic	ANO6	NA	NA	NA	NA	NA	0.009	0.475	TG
ETV6	12-11655600-C-T	12	rs1894330	Intronic	ETV6	NA	NA	NA	NA	NA	0.005	0.337	TC
ETV6	12-11766473-C-T	12	rs3825083	Intronic	ETV6	NA	NA	NA	NA	NA	0.006	0.167	TC
ETV6	12-11767464-G-A	12	rs60587284	Intronic	ETV6	NA	NA	NA	NA	NA	0.005	0.169	AG
ETV6	12-11773615-C-T	12	rs61921814	Intronic	ETV6	NA	NA	NA	NA	NA	0.002	0.030	TC
STAB2	12-103741305-G-A	12	rs3844213	Intronic	STAB2	NA	NA	NA	NA	NA	0.004	0.238	GA
STAB2	12-103594533-T-C	12	rs703597	Intronic	STAB2	NA	NA	NA	NA	NA	0.005	0.227	CT
TBK1	12-56356420-C-T	12	rs2066819	Intronic	TBK1	NA	NA	NA	NA	NA	0,000	0,065	TC
ABCC4	13-95210855-T-G	13	rs16950758	Intronic	ABCC4	NA	NA	NA	NA	NA	0.053	0.006	GT
NBEA	13-35599778-A-G	13	rs78478047	Intronic	NBEA	NA	NA	NA	NA	NA	0.002	0.022	GA
NBEA	13-35045248-A-G	13	rs9543121	Intronic	NBEA	NA	NA	NA	NA	NA	0.003	0.171	GA
SLC7A7	14-22826823-G-A	14	rs56252908	Intronic	SLC7A7	NA	NA	NA	NA	NA	0.007	0.115	AG
VIPAS39	14-77429241-G-A	14	rs116854785	Intronic	VIPAS39	NA	NA	NA	NA	NA	0.012	0.010	AG
BLOC1S6	15-45600721-G-A	15	rs117544584	Intronic	AC090527.2, BLOC1S6	NA	NA	NA	NA	NA	0.001	0.033	AG
TRPM7	15-50565263-T-G	15	rs1986073	Intronic	TRPM7	NA	NA	NA	NA	NA	0.007	0.212	GT
VKORC1	16-31094233-A-C	16	rs2884737	ncRNA exonic; splicing	AC135050.7; VKORC1(ENST00000394971.7:exon1:c.267 + 2T > G)	Drug response	Warfarin response—Dosage	Reviewed by expert panel	NA	NA	0.002	0.256	CA
GRN	17-44331964-T-C	17	rs77316809	Downstream	AC003043.1	NA	NA	NA	NA	NA	0.083	0.008	CT
ITGB3	17-47302881-G-A	17	rs13306488	Intronic	AC068234.1, ITGB3	NA	NA	NA	NA	NA	0.000	0.003	AG
CD70	19-6593591-C-T	19	rs344595	Intronic	CD70	NA	NA	NA	NA	NA	0.005	0.118	CT
SLC44A2	19-10628918-T-G	19	rs11670384	Intronic	SLC44A2	NA	NA	NA	NA	NA	0.001	0.203	GT
ADA	20-44631548-T-C	20	rs73113339	Intronic	ADA	NA	NA	NA	NA	NA	0.001	0.027	CT
RUNX1	21-34990282-T-C	21	rs2242878	Intronic	RUNX1	NA	NA	NA	NA	NA	0.007	0.179	TC
RUNX1	21-35595917-G-A	21	rs4817730	Intronic	RUNX1	NA	NA	NA	NA	NA	0.006	0.125	GA
RUNX1	21-34902639-T-C	21	rs6417685	Intronic	RUNX1	NA	NA	NA	NA	NA	0.004	0.095	TC
RUNX1	21-35179264-T-G	21	rs9977362	Intronic	RUNX1	NA	NA	NA	NA	NA	0.010	0.669	CT
IFNAR2	21-33186096-A-C	21	rs12626404	Intergenic	LINC01548(dist = 15,447), IFNAR2(dist = 43,805)	NA	NA	NA	NA	NA	0,000	0,259	CA
IFNAR2	21-33209547-A-G	21	rs2834146	Intergenic	LINC01548(dist = 38,898), IFNAR2(dist = 20,354)	NA	NA	NA	NA	NA	0,003	0,246	AG
IFNAR1	21-33318683-G-A	21	rs62228028	Intergenic	IL10RB(dist = 8,496), IFNAR1(dist = 5,746)	NA	NA	NA	NA	NA	0,002	0,077	AG
HPS4	22-26483125-G-A	22	rs9620611	Intronic	HPS4	NA	NA	NA	NA	NA	0.005	0.288	AG
MTHFR	1-11796321-G-A	1	rs1801133	exonic	MTHFR	Drug response	Neoplasm of stomach; Gastrointestinal stromal tumor; Thrombophilia due to thrombin defect; Homocystinuria due to methylene tetrahydrofolate reductase deficiency; Neural tube defects, folate-sensitive; MTHFR deficiency, thermolabile type; Homocystinuria due to MTHFR deficiency; carboplatin response—Efficacy; cyclophosphamide response—Toxicity/ADR; methotrexate response—Dosage, Efficacy, Toxicity/ADR; not provided	ENST00000376486.3, ENST00000376583.7, ENST00000376585.6, ENST00000376590.8, ENST00000376592.6, ENST00000423400.7, ENST00000641407.1, ENST00000641446.1	Deleterious	Probably damaging	0,079	0,365	AA
F11	4-186271327-T-C	4	rs2036914	Intronic	F11	Benign	not provided	criteria provided, single submitter	NA	NA	0,320	0,471	TT
F11	4-186269656-A-G	4	rs4253405	Intronic	F11	NA	NA	NA	NA	NA	0,112	0,400	GG
TLR3	4-186125608-C-T	4	rs6849187	Intronic	FAM149A	NA	NA	NA	NA	NA	0,490	0,487	CC
TLR3	4-186089972-C-T	4	rs6857595	Intergenic	TLR3(dist = 1,903), FAM149A(dist = 14,447)	NA	NA	NA	NA	NA	0,324	0,255	TT
IFNW1	9-21114676-G-A	9	rs7852828	Intergenic	IFNB1(dist = 36,734), IFNW1(dist = 25,538)	NA	NA	NA	NA	NA	0,438	0,395	AA
TBK1	12-64582269-C-A	12	rs1245035	Intronic	RASSF3	NA	NA	NA	NA	NA	0,431	0,370	CC
TBK1	12-64590589-C-T	12	rs1520765	Intronic	RASSF3	NA	NA	NA	NA	NA	0,484	0,438	CC
TICAM1	19-4800091-C-A	19	rs4807643	UTR3	FEM1A(ENST00000269856.4:c.*6227C > A)	NA	NA	NA	NA	NA	0,134	0,360	CC
TICAM1	19-4804512-T-C	19	rs8102626	Intergenic	FEM1A(dist = 3,239), TICAM1(dist = 11,420)	NA	NA	NA	NA	NA	0,231	0,180	CC
IFNAR2	21-33245645-A-T	21	rs2252650	Intronic	AP000295.1, IFNAR2	NA	NA	NA	NA	NA	0,224	0,333	AA

SNPs, single-nucleotide polymorphism; VCF, variant call format; Chr, chromosome; SIFT, sorting intolerant from tolerant; AFR, African population; EUR, European population; 1KGP, 1000 Genomes Project; MAF, minor allele frequency; NA, not available.

In addition to the search for rare variants, a second strategy was employed. We performed a screening of common mutations in the European or African populations that were in homozygosity in the patient. We assessed classical hereditary thrombophilia-associated mutations: Factor V Leiden G1691A (rs6025), Factor II G20210A (rs1799963), and methylenetetrahydrofolate reductase (*MTHFR*), C677T (rs1801133) and A1298C (rs1801131), in addition to distinct mutations on *F11* and genes related to type I IFN. The patient was homozygous for the missense *MTHFR* variant c.665C > T chr1-11856378 G > A p.Ala222Val NM_005957.5 rs1801133, with clinical relevance for methotrexate drug response, with a general population frequency of approximately 0.3. His medical records from January 2021 and 2008 showed normal levels of folic acid and homocysteine, respectively. Also, he was not on vitamin B12 supplementation or had ever had hemolysis. Furthermore, he was homozygous for the variants in *FXI* rs2036914 and rs4253405; in *TLR3* rs6849187 and rs6857595; in *IFNW1* rs7852828; in *TBK1/RASSF3* rs1245035 and rs1520765; in *TICAM1* rs4807643 and rs8102626; and in *IFNAR2* rs2252650. Besides rs2036914, classified as benign, none of these variants have been described as associated with clinical diseases (Table 3 in gray).

## Discussion

VITT is a rare but life-threatening disease described after the COVID-19 vaccination rollout with the adenoviral platform (2). The regulatory agencies use thrombosis with thrombocytopenia syndrome (TTS) as a descriptive term for VITT, not necessarily caused by vaccination (10). Herein, we describe a patient who developed a clinical condition consistent with the Level 1 TTS Brighton Collaboration case definition after the first dose of ChAdOx1 nCoV-19 (11). Our patient was one of the 39 VITT cases described in Brazil after primary vaccination in 2021 (12). According to the UK Expert Hematology Panel published by Pavord et al. (2), he was also classified as a definite VITT case. This series of 220 VITT British patients identified platelet counts of less than 30,000/mm^3^ and the presence of intracranial hemorrhage as being independently associated with death, with 73% mortality if they coexist (2). Our patient presented both poor prognostic factors and succumbed despite proper healthcare assistance.

Differences in VITT incidence worldwide support distinct genetic ancestries on pathogenesis, although other explanations, such as underreporting and health systems inequalities, should be addressed. The highest incidence was reported in Norway after ChAdOx1 nCoV-19, with five cases among 130,000 individuals, suggesting an incidence of 1 in 26,000 (13). In the United States, the VAERS surveillance system identified 54 cases of TTS from among over 14 million recipients of Ad26.COV2.S, for an incidence of 3.8 per million (approximately 1 in 263,000) (14). In addition to age below 50 years and first exposure to the COVID-19 adenovirus vaccine within 30 days, VITT risk factors are unknown and seem to differ from the traditional prothrombotic conditions. Unlike HIT, VITT is caused by monoclonal or oligoclonal anti-PF4 antibodies (7). This finding also indicates a potential role for the genetic predisposition in VITT pathophysiology. Thus, further studies characterizing anti-PF4 antibody–producing cells are needed.

The effect of *MTHFR* variants on thrombotic risk is controversial. Recent guidelines state that *MTHFR* polymorphisms should not be a part of inherited thrombophilia testing due to a lack of clinical evidence (15, 16). However, a meta-analysis based on case-control studies found that the rs1801133 *MTHFR* C677T polymorphism—the same identified in our patient—could increase ischemic stroke susceptibility in Asian, male, and young-middle age populations (17). Another meta-analysis enrolled 99 genetic association studies, including Brazilians, concluded that the *MTHFR* rs1801133 polymorphism might be implicated in developing deep vein thrombosis and pulmonary embolism in non-VITT patients and may serve as a potential biological marker for venous thromboembolism in Caucasians, East Asians, and West Asians (18). Yet, the frequency of *MTHFR* polymorphisms in VITT is unexplored. A heterozygous *MTHFR* C677T rs1801133 variant has been identified in an Italian patient with cerebral sinus thrombosis with thrombocytopenia after COVID-19 vaccination and increased levels of homocysteine and folate deficiency (19). This description was a probable VITT case, given the lack of anti-PF4 positivity. Another paper from Germany has reported two women presenting with cerebral sinus vein thrombosis after the ChAdOx1 vaccine, each carrying an *MTHFR* variant (heterozygous A1298C and homozygous *MTHFR* C677T variant) (20).

The *F11* variant rs116667976 found in heterozygosity in the patient is classified as likely benign by ACMG and presented as having conflicting interpretations of pathogenicity in the ClinVar database. It has been selected as a potentially functional mutation in thrombosis without functional analysis available (21). The same SNP was described in 2 out of 49 women with heavy menstrual bleeding. However, functional data are still missing for assessing the involvement of this very rare variant in thrombotic diseases (22). The other *F11* variants that were presented in two alleles of the patient, rs2036914 and rs4253405, African and European, have been evaluated in studies of percutaneous coronary intervention and venous thrombosis, respectively, but the genotype found in the patient from our study has not been described to be associated with any disease (23). The high allele frequency of these SNPs makes it difficult to find any associations.

Type I IFNs are induced by exposure of cells to pathogen-associated molecular patterns (PAMPs) detected by receptors like toll-like receptors (TLRs). By distinct mechanisms, type I IFN signaling leads to inflammasome activation, pyroptosis, and, lately, the release of proinflammatory molecules and prothrombotic mediators, like TF, initiating the extrinsic coagulation pathway (24). Due to the involvement of type I IFN response to thrombotic processes, we also evaluated SNPs in the related genes. We found eight variants in homozygosity in the patient. However, none of them are found in exons or have been studied for thrombotic diseases.

Early recognition and treatment are essential for a favorable outcome in VITT. Risk factors are still poorly understood. We described a case report of VITT in an individual harboring a benign rs1801133 homozygous variant in *MTHFR* and the rs116667976, rs2036914, and rs4253405 in the *F11*, apart from homozygous mutations in *IFNAR2*, *IFNW1*, *TBK1*, *TICAM1*, and *TLR3* genes without reported clinical significance. Although these findings could favor a genetic predisposition, most of the variants found are frequent, and further genomic research is needed to establish a causal association.

## Patient perspective

This work is of value for alerting healthcare professionals to the early signs and symptoms of VITT and adding information about a possible genetic background related to the disease development.

## Data Availability

The original contributions presented in the study are included in the article/supplementary materials, further inquiries can be directed to the corresponding author.
